# Harnessing the Power of Light: The Synergistic Effects of Crystalline Carbon Nitride and Ti_3_C_2_T_x_ MXene in Photocatalytic Hydrogen Production

**DOI:** 10.1002/gch2.202300235

**Published:** 2024-05-11

**Authors:** Khai Jie Wong, Joel Jie Foo, Tan Ji Siang, Valerine Khoo, Wee‐Jun Ong

**Affiliations:** ^1^ School of Energy and Chemical Engineering Xiamen University Malaysia Selangor Selangor Darul Ehsan 43900 Malaysia; ^2^ Center of Excellence for NaNo Energy & Catalysis Technology (CONNECT) Xiamen University Malaysia Selangor Selangor Darul Ehsan 43900 Malaysia; ^3^ State Key Laboratory of Physical Chemistry of Solid Surfaces College of Chemistry and Chemical Engineering Xiamen University Xiamen 361005 China; ^4^ Gulei Innovation Institute Xiamen University Zhangzhou 363200 China; ^5^ Shenzhen Research Institute of Xiamen University Shenzhen 518057 China

**Keywords:** carbon nitride, crystallinity, hydrogen production, MXene, photocatalyst

## Abstract

Photocatalytic hydrogen evolution is an environmentally friendly means of energy generation. Although g‐C_3_N_4_ possesses fascinating features, its inherent shortcomings limit its photocatalytic applications. Therefore, modifying the intrinsic properties of g‐C_3_N_4_ and introducing cocatalysts are essential to ameliorate the photocatalytic efficiency. To achieve this, metal‐like Ti_3_C_2_T_x_ is integrated with crystalline g‐C_3_N_4_ via a combined salt‐assisted and freeze‐drying approach to form crystalline g‐C_3_N_4_/Ti_3_C_2_T_x_ (CCN/TCT) hybrids with different Ti_3_C_2_T_x_ loading amounts (0, 0.2, 0.3, 0.4, 0.5, 1, 5, 10 wt.%). Benefiting from the crystallization of CN, as evidenced by the XRD graph, and the marvelous conductivity of Ti_3_C_2_T_x_ supported by EIS plots, CCN/TCT/Pt loaded with 0.5 wt.% Ti_3_C_2_T_x_ displays an elevated H2 (2) should be subscripted evolution rate of 2651.93 µmol g^−1^ h^−1^ and a high apparent quantum efficiency of 7.26% (420 nm), outperforming CN/Pt, CCN/Pt, and other CCN/TCT/Pt hybrids. The enhanced performance is attributed to the synergistic effect of the highly crystalline structure of CCN that enables fleet charge transport and the efficient dual cocatalysts, Ti_3_C_2_T_x_ and Pt, that foster charge separation and provide plentiful active sites. This work demonstrates the potential of CCN/TCT as a promising material for hydrogen production, suggesting a significant advancement in the design of CCN heterostructures for effective photocatalytic systems.

## Graphical abstract

To address the limitations of g‐C_3_N_4_, crystalline carbon nitride/Ti_3_C_2_T_x_ MXene (CCN/TCT) hybrids are ingeniously devised via a combined salt‐assisted and freeze–drying method. The optimal CCN/TCT/Pt hybrid demonstrates exceptional performance in photocatalytic hydrogen evolution attributing to the synergistic effect of the CCN with highly crystalline structure and the efficient dual cocatalysts, Ti_3_C_2_T_x_ and Pt.

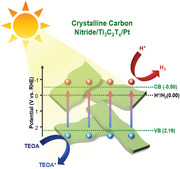

## 1 Introduction

The rapid population growth and the flourishing development of human society have led to tremendous consumption of fossil energy, which in turn induces energy shortages and environmental issues.^[^ ^1^ ^]^ In this regard, the exploration of sustainable and renewable energy has invigorated the growing attention of society over the past decades.^[^ ^2^ ^]^ Light‐driven H_2_ production is an attractive and sought‐after strategy for the transformation of inexhaustible solar energy into clean and renewable chemical fuels.^[^ ^3^ ^]^ Polymeric carbon nitride (CN) has emerged as a promising candidate for photocatalytic H_2_ evolution ascribing to its merit of well‐matched electronic structure, outstanding stability, nontoxicity, and cost‐effective synthesis.^[^ ^4^ ^]^ The basic building blocks of CN are tri‐s‐triazine units, where the three nitrogen atoms and three carbon atoms are arranged in a hexagonal network similar to graphene.^[^ ^1^ ^]^ The layers, held together by weak van der Waals, stack on top of each other, forming a bulk material. Despite exhibiting fascinating properties, pristine CN exhibits weak photocatalytic activity as a result of the rapid recombination efficiency of photoactivated electron‐hole pairs, which is caused by the inherent π‐conjugated electronic system.^[^ ^4^, ^5^ ^]^

Typically, CN nanosheets are prepared via thermal polymerization of precursors, such as urea, dicyandiamide, and thiourea, where the resultant CN suffers from low crystallinity due to incomplete condensation.^[^ ^6^ ^]^ The disordered structure or phase defects would serve as electron traps that restrict the intra‐ or/and interlayer charge transfer to a certain extent, thus leading to elevated charge recombination and limiting the photocatalytic performance.^[^ ^7^ ^]^ Therefore, many efforts have been dedicated to explore effective modification strategies, including structural engineering,^[^ ^8^ ^]^ defect engineering,^[^ ^9^ ^]^ and heterojunction engineering.^[^ ^10^ ^]^ Aside from external activity promotion, there is also much room for improvement in the inherent characteristics of CN, where the ameliorated intrinsic activity will benefit the external promotion in return.^[^ ^11^ ^]^ Thus, in pursuit of enhancing the intrinsic properties and further improving the photoactivity of g‐C_3_N_4_, researchers endeavored to mitigate the structural defects of the material by reinforcing its crystallinity.^[^ ^11^ ^]^ This was achieved through the introduction of metal cations, intercalated within the structure and stabilized by the surrounding nitrogen bridge atoms, thereby forming well‐defined structural pores.^[^ ^12^ ^]^ Crystalline carbon nitride (CCN) displays great superiority as it retains the merits of CN while exerting ameliorated charge segregation efficiency. CCN consists of well‐ordered, periodic structures of carbon and nitrogen atoms. Nonetheless, depending on the molten salts used in preparing the CCN, different alkali metals will be incorporated within the structure of CCN, thus giving rise to different effects on the crystal structure, morphology, and optical characteristics of the CCN.^[^ ^6^, ^13^ ^]^ CN is generally crystallized in the presence of molten salts (also known as ionothermal method)^[^ ^14^ ^]^ or solid salts,^[^ ^15^ ^]^ where the salts assist in inducing more complete polymerization of the in‐plane heptazine‐based melon chains, which subsequently brings about bolstered photocatalytic H2 evolution performance. Molten salts are capable of dissolving monomers and intermediates, making them a suitable liquid reaction medium that can remain stable at high temperatures and operate beyond the upper limits of organic solvents.^[^ ^16^ ^]^ This property thus enables the polycondensation process of carbon materials that generally necessitate high temperatures. Different combinations of salts (for example, LiCl/KCl, NaCl/KCl) will lead to varying melting points of the molten salts, which will subsequently affect the polymerization process and the ensuing CCN.^[^ ^14^, ^16^ ^]^

In addition to the optimization of CN's inherent properties, incorporating another semiconductor or metal into CN to form a heterostructure is also one of the effective methods to facilitate charge segregation for enhanced photocatalysis.^[^ ^17^ ^]^ In view of this, explosive interest has been attended to introduce a variety of cocatalysts into CN for accelerating charge transport and separation.^[^ ^4^ ^]^ In recent years, MXenes, a family of 2D transition metal carbides, nitrides, and carbonitrides, which are commonly obtained from selective etching and exfoliation of their parent MAX phases, have received growing attention attributed to their multifarious intriguing and distinctive characteristics, such as remarkable conductivity, suitable Fermi level, ease of heterojunction construction, and numerous surface terminations.^[^ ^18^ ^]^ On the one hand, the extraordinary metallic conductivity and appropriate Fermi level bestow MXenes the ability to function as an electron reservoir, where the formation of Schottky junction between MXenes and *n*‐type semiconductors are beneficial for the efficient charge transport and segregation, and hence the boosted photoactivity.^[^ ^19^ ^]^ On the other hand, the abundant functional groups on the surface of MXenes drastically raise the density of active sites, which is conducive to the adsorption and activation of the desired reactant molecules and the following redox reaction.^[^ ^20^ ^]^ Meanwhile, the firm interface established between MXenes and semiconductors favors the charge migration across the heterojunction interface, which dramatically enhances photocatalytic capacity.^[^ ^21^ ^]^ Stemming from all the excellent features of MXenes, MXenes exert striking potential in photocatalytic reactions.

Taking into account the unique characteristics of MXenes, Ti_3_C_2_T_x_‐based MXenes have been extensively used for enhancing photocatalytic activity in recent years.^[^ ^22^ ^]^ For example, Liu et al. constructed a g‐C_3_N_4_/Ti_3_C_2_T_x_/Pt heterojunction for photocatalytic hydrogen evolution and contaminant degradation, where the as‐synthesized catalysts achieved an H_2_ production rate of 1948 µmol g^−1^ h^−1^.^[^ ^23^ ^]^ Although the photocatalytic performance of CN/TCT has been improved due to the formation of heterojunction and the presence of TCT serving as effective cocatalysts, the potential of CN/TCT has not been fully exploited due to the amorphous structure of CN that restricts the efficient charge transport and separation, thereby limiting the photoactivity. In this regard, coupling MXene with CN with high crystallinity is a propitious strategy for surmounting this bottleneck. Inspired by the significance of the crystallinity of CN and the astounding attributes of MXenes, we have successfully devised CCN/TCT MXene heterostructure and investigated their use as photocatalysts for hydrogen production. The as‐prepared CCN/TCT hybrids displayed a robust and significantly enhanced hydrogen production rate in comparison with pristine CN and CCN. In particular, the hydrogen generation rate of the optimal sample, in this case, CCN loaded with 0.5 wt.% Ti_3_C_2_T_x_ was 2651.93 µmol g^−1^ h^−1^, which was 43.48‐ and 1.09‐fold higher than that of bare CN and CCN. The ameliorated charge transfer dynamics and enhanced photoconversion efficiency originated from the synergistic effects of the highly crystalline structure of CCN, distinguished conductivity as well as abundant active sites offered by Ti_3_C_2_T_x_ and Pt.

## 2 Experimental Section

### 2.1 Materials

All chemicals were analytical grade and used as received without further purification. Urea (99%), ethanol (99%), Nafion (99%), HCl (99%), LiF (99%), NaCl (99%), KCl (99%), KOH (99%), and triethanolamine (99%) were purchased from Sigma–Aldrich. Ti_3_AlC_2_ (99%) was purchased from Forsman Scientific (Beijing). Deionized (DI) water was used throughout the experiments.

### 2.2 Synthesis of Amorphous g‐C_3_N_4_ Nanosheets (CN)

Urea of 6 g was placed in a crucible with a lid and heated to 550 °C at a rate of 10 °C min^−1^ in air atmosphere and maintained at 550 °C for 2 h. Upon cooling down, the as‐acquired g‐C_3_N_4_ was ground to obtain it in powder form.

### 2.3 Synthesis of Crystalline g‐C_3_N_4_ Nanosheets (CCN)

CCN was prepared via the ionothermal method. Initially, 10 g of urea was mixed and ground with 10 g NaCl, 9.5 g KCl, and 0.5 g KOH at a mass ratio of 20: 20: 19: 1 (urea: NaCl: KCl: KOH) for 15 min in a mortar. Subsequently, the mixture was transferred to a crucible with a cover. The mixture was heated to 550 °C at a rate of 10 °C min^−1^ in the air atmosphere and maintained at 550 °C for 2 h. Upon cooling down to room temperature, the product was washed with de‐ionized water at a temperature of ≈60 °C for several times to remove the residual salts. Finally, the obtained powder was dried overnight at 60 °C.

### 2.4 Synthesis of Ti_3_C_2_T_x_

Ti_3_C_2_T_x_ was prepared by first dispersing 2 g LiF in 40 mL 9 m HCl solution and stirring for 5 min, followed by the slow addition of 2 g Ti_3_AlC_2_ powder into the solution over a 5 min period, and stirred at 40 °C at 60 h. The mixtures were then washed with distilled water and centrifuged at 3500 rpm for 5 min until pH reached ≈6. Next, the mixture was dried overnight at 60 °C under vacuum conditions.

### 2.5 Synthesis of Crystalline g‐C_3_N_4_/Ti_3_C_2_T_x_

**Figure 1:**
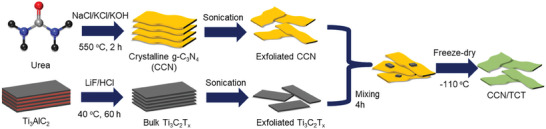
Schematic diagram of the synthesis of CCN/TCT.

CCN of 100 mg was dissolved in 20 mL DI water and ultrasonicated for 1 h. 0.5 mg Ti_3_C_2_T_x_ was added into 20 mL DI water and ultrasonicated for 1 h. The Ti_3_C_2_T_x_ solution was then added dropwise to the CCN suspension over 5 min, and the mixture was stirred for 4 h to achieve uniform suspension. The mixture was washed one time and freeze–dried to obtain the composite, which was denoted as CCN/TCT‐0.5. Later, a series of CCN/Ti_3_C_2_T_x_ heterostructures were prepared by varying the mass of Ti_3_C_2_T_x_, based on the designed Ti_3_C_2_T_x_ to CCN mass ratio: 0.2, 0.3, 0.4, 1, 5, 10 wt.%, which were denoted as CCN/TCT‐1, CCN/TCT‐5 and CCN/TCT‐10, respectively. **Figure** **1** displays the synthetic process of CCN/TCT. CCN was prepared by molten salt‐assisted polymerization method with urea acting as the precursor, where NaCl, KCl, and KOH were added to assist in the formation of nanosheets and crystallization, respectively. Meanwhile, the multi‐layered Ti_3_C_2_T_x_ was obtained through the etching of Ti_3_AlC_2_ by LiF and HCl. The bulk Ti_3_C_2_T_x_ was subsequently exfoliated into few‐layer ultrathin Ti_3_C_2_T_x_ nanosheets under sonication. CCN/TCT was acquired upon mixing for 4 h and freeze–drying.

### 2.6 Characterization

The crystalline phases, morphologies, and microstructures of the samples were characterized by X‐ray measurement using PANalytical X'Pert Pro with Cu Kα as the radiation source (λ = 1.54 Å), scanning electron microscopy (SEM), and transmission electron microscopy (TEM), respectively. The SEM images were obtained with a field‐emission microscope by a Carl Zeiss GeminiSEM 500‐70‐22 instrument whereas the TEM was carried out using FEI Tecnai G2 20 S‐TWIN system. The Fourier‐transform infrared (FTIR) spectra were measured on PerkinElmer FTIR Frontier in the 4000 to 400 cm^−1^ range. The atomic environment and chemical composition were analyzed using an X‐ray photoelectron spectroscopy (XPS, ESCALab220i‐XL) with a monochromatic Al Kα X‐ray source. The C 1s peak at 284.9 eV was used as an internal standard. The optical properties were studied by UV–vis diffuse reflectance spectrophotometry (UV–vis DRS) in a Jasco V‐750 spectrophotometer with clean fluoride tin oxide (FTO) glass as the reflectance standard.

### 2.7 Photoelectrochemical (PEC) Measurements

The photo‐electrochemical experiments were carried out in a standard three‐electrode system at room temperature (27 °C) by an electrochemical workstation equipped with a 300 W Xe lamp. The reference, counter, and working electrodes were Ag/AgCl, Pt wire, and FTO coated with catalyst, respectively. The working electrode was prepared by dropping 10 µL suspension onto the glass conductive side of fluoride tin oxide (FTO) with (1 cm × 1 cm) area. The detailed steps were as follows: FTO glass was rinsed using ethanol several times to clean the surface. The suspension was prepared by dispersing 5 mg of catalyst in a mixture of 30 µL Nafion and 125 µL absolute ethanol solution and stirred until a homogenous solution was obtained. By using a measuring micropipette, 10 µL of the sample was loaded on an FTO clean glass electrode with (1 cm × 1 cm) coated area and dried naturally. All these tests were performed in 0.1 m Na_2_SO_4_ aqueous solution (electrolyte). The Mott–Schottky (MS) plots were measured in the voltage range of −1 to 1 V with the frequency of 500, 750, and 1000 Hz. The electrochemical impedance spectra (EIS) were measured under open‐circuit voltage with frequencies ranging from 10^−2^ to 10^5^ Hz (AC amplitude of 10 mV) in a 0.1 m Na_2_SO_4_ solution.

### 2.8 Photocatalytic Performance Evaluation

**Figure 2:**
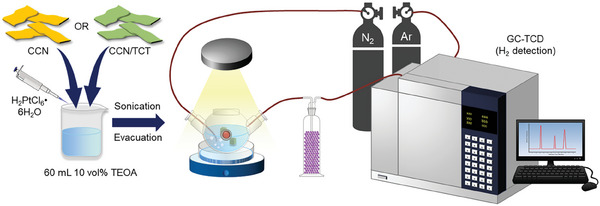
Photocatalytic performance evaluation of carbon nitride and hybrid photocatalysts for HER.

The photocatalytic HER was carried out in a quartz reactor with a closed gas circulation system (**Figure** **2**). A water bath was used to maintain the reactor at a fixed temperature (25 °C). A 300 W Xe‐lamp with an ultraviolet cut‐off filter (λ ≥ 420 nm) was used as the light source. 20 mg of the as‐prepared samples were dispersed in 60 mL 10 vol% TEOA aqueous solution with 0.534 mL 0.995 g L^−1^ H_2_PtCl_6_•6H_2_O and sonicated for 30 min in the dark to form uniform dispersion. Next, the whole reaction system was stirred and evacuated using N_2_ gas to remove air and reach adsorption and desorption equilibrium between catalysts and TEOA solution in a closed gas circulation system for 30 min, and then sealed. The reaction was carried out (300 W Xe‐lamp, λ ≥ 420 nm) for 4 h. The evolved H_2_ amount was determined by gas chromatography (Agilent GC‐7890B, MolSieve 5A Column, Ar as carrier gas) every 30 min. The apparent quantum efficiency (AQE) at 420 nm was calculated under the same experimental conditions using the following equation:

(1) $$AQE=\frac{2\times number\;of\;evolved\;H_{2}\;molecules}{number\;of\;incident\;photons}\times 100\%$$

The long‐term stability test was performed under identical conditions to that of the photocatalytic HER.

## 3 Results and Discussion

### 3.1 Morphology and Microstructure

**Figure 3:**
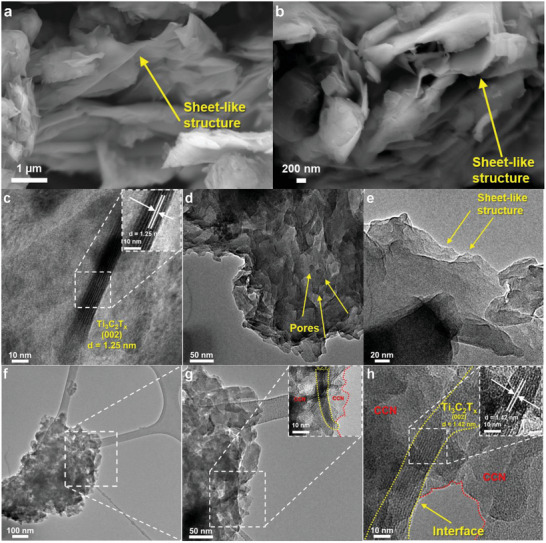
Field‐emission scanning electron microscopy (FESEM) images of Ti_3_C_2_T_x_ under magnification of a) 1 µm and b) 200 nm. TEM and high‐resolution TEM (HRTEM) images of c) Ti_3_C_2_T_x_, d) e) CCN, and f) g) h) CCN/TCT‐0.5. Insets of c), g), and h) show the zoomed‐in images of Ti_3_C_2_T_x_ and CCN/TCT‐0.5, respectively.

The morphology and structure of the Ti_3_C_2_T_x_ were first characterized by FESEM. As indicated in **Figure** **3**a and Figure 3b, the Ti_3_C_2_T_x_ after etching treatment exhibits a multi‐layered structure due to the removal of the Al layer,^[^ ^24^ ^]^ where the sheet‐like structure is observed at the edge of the MXene. Upon sonication, the exfoliated ultrathin sheet‐like structure is expected, which is beneficial in aiding the efficient mobility of photoexcited charge carriers.^[^ ^25^ ^]^ During the fabrication process of CCN/TCT, CCN and TCT self‐assemble due to various forces, such as van der Waals, and form a composite structure in a solution during the 4 h stirring process. When the self‐assembled structures are frozen and subjected to freeze–drying, the frozen solvent undergoes sublimation without disrupting the ordered arrangement structure, leaving the final product in a dry state while maintaining the structure and arrangement achieved during self‐assembly.

To gain further insights into the structure of Ti_3_C_2_T_x_, CCN, and CCN/TCT hybrid, the TEM and HRTEM measurements are performed, and their images are presented in Figure 3c–g. As depicted in Figure 3c, Ti_3_C_2_T_x_ exerts a multi‐layered sheet‐like structure, which is aligned with the FESEM images of Ti_3_C_2_T_x_ (Figure 3a,b). The lattice fringe of Ti_3_C_2_T_x_ appears notably in the inset of Figure 3c, where the lattice spacing of 1.25 nm corresponds to the characteristic (002) plane.^[^ ^26^ ^]^ Meanwhile, the several layered thick structures with holey texture defects with pore sizes of 9.2–16.3 nm displayed in Figure 3d and Figure 3e belong to CCN. After introducing 0.5 wt.% Ti_3_C_2_T_x_ into CCN, the morphology of CCN is nearly unchanged (Figure 3f,g), since the loading amount of Ti_3_C_2_T_x_ is considerably low. The TEM images of the CCN/TCT‐0.5 composite (Figure 3g,h) depict that the porous nanosheets and nonporous nanosheets are stacked closely, where a distinct interface between them is observed. The commendable interaction between CCN and Ti_3_C_2_T_x_ is further ascertained by the inset of Figure 3h, where the clear lattice fringe of Ti_3_C_2_T_x_ is detected in the 2D/2D layered arrangement CCN/TCT‐0.5 heterostructures, which is similar to the previous works on g‐C_3_N_4_/Ti_3_C_2_T_x_.^[^ ^27^ ^]^

### 3.2 Structure and Composition

**Figure 4:**
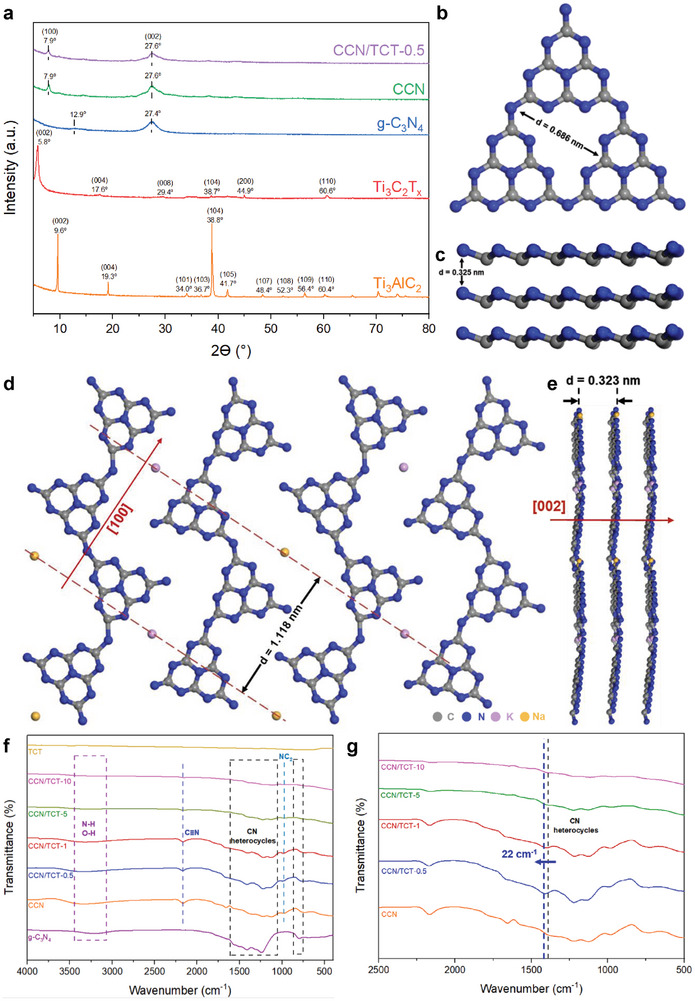
a) XRD pattern of CCN/TCT‐0.5, CCN, g‐C_3_N_4_, Ti_3_C_2_T_x_, and Ti_3_AlC_2_. Postulated b) in‐plane and c) interlayer crystal structures in g‐C_3_N_4_. Postulated d) in‐plane and e) interlayer crystal structures in CCN. f) FTIR of TCT, g‐C_3_N_4_, CCN, and CCN/TCT hybrids with varying Ti_3_C_2_T_x_ loading amounts. g) Zoomed‐in FTIR of CCN and CCN/TCTs.

In order to confirm the formation of Ti_3_C_2_T_x_ as well as CCN, the composition and crystallinity of the samples, namely Ti_3_C_2_T_x_, Ti_3_AlC_2_, g‐C_3_N_4_, CCN, and CCN/TCT‐0.5, were characterized by X‐ray diffraction (XRD) analysis. From **Figure** **4**a, the sample with intense peaks is indexed to the Ti_3_AlC_2_ MAX phase. After LiF/HCl etching treatment, the diffraction peak of (002) plane at 9.6° is broadened and shifted to a lower angle of 5.8°, indicating an expansion of the *c* lattice parameter from 0.921 to 1.523 nm, which is consistent with the HRTEM image of Ti_3_C_2_T_x_ (Figure 3h).^[^ ^28^ ^]^ The extended lattice spacing is attributed to the replacement of the Al layer with functionalities such as ─OH and ‐F.^[^ ^28^ ^]^ Meanwhile, the disappearance of the most intense peak of (104) plane at 38.8°, which corresponds to the characteristic peak of Ti_3_AlC_2_, implies the complete removal of the Al layer and the successful transformation of Ti_3_AlC_2_ to Ti_3_C_2_T_x_.^[^ ^29^ ^]^ Moreover, the overall peak intensities of Ti_3_C_2_T_x_ are weaker than that of Ti_3_AlC_2_, which originated from the thinner layered structure of Ti_3_C_2_T_x_.^[^ ^30^ ^]^

Besides, as implied in Figure 4a, the amorphous sample of g‐C_3_N_4_ shows two typical diffraction peaks at 12.9° and 27.4°, ascribing to the (100) in‐plane distance of the nitrogen‐linked heptazine units and (002) interlayer π–π stacking, respectively.^[^ ^31^ ^]^ For crystalline carbon nitride, the (100) peak shifts to 7.9°, indicating an extended in‐plane arrangement distance from 0.686 to 1.118 nm. The enlarged intralayer packing of polymeric melon units stems from the presence of Na and K atoms with larger atomic radii than that of C and N within the heptazine structure.^[^ ^32^ ^]^ This depicts that the CCN exerts enhanced crystallinity with an unfolded in‐plane framework that is associated with sufficient condensation of the conjugated network.^[^ ^33^ ^]^ Whereas the main peak of (002) facet moves from 27.4° to 27.6°, corresponding to the narrowed interlayer spacing from 0.325 to 0.323 nm.^[^ ^32^ ^]^ The reduced interlayer distance is attributed to the introduction of Na and K atoms, where the intercalated metal ions disrupt the ordered periodic stacking of the carbon nitride structure and strengthen the interlayer coupling by coordinating with adjacent N atoms, thereby resulting in a compacted packing and enhanced crystallinity of the melon framework.^[^ ^25^, ^34^ ^]^ Overall, the close interlayer packing is advantageous to the transfer of photogenerated charge carriers between the layers, and the subsequent photocatalytic activity. Furthermore, the (100) peaks become stronger and narrower, revealing an improved crystallinity of CCN as compared to g‐C_3_N_4_.^[^ ^34a^ ^]^ In addition, the full width at half‐maximum (FWHM) of the (002) peak of the CCN (2.66°) sample is narrowed compared to that of g‐C_3_N_4_ (2.71°), which again signifies the ameliorated crystallinity of CCN over bulk g‐C_3_N_4_. Based on the XRD results and literature studies,^[^ ^13^, ^15^ ^]^ the hypothetical in‐plane and interlayer structures of g‐C_3_N_4_ are signified in Figure 4b,c, respectively, whereas Figure 4d,e displays the postulated structures of CCN. As a whole, the higher crystalline degree of CCN provides a higher separation and transfer efficiency of photoexcited charges, which is propitious to the enhancement of photocatalytic performance,^[^ ^35^ ^]^ and this statement will be affirmed by the EIS plots in Section 3.4. It should be noted that the CCN/TCT composite presented similar XRD patterns to that of CCN (Figure 4a), manifesting that the condensation of melon units is not influenced by the existence of Ti_3_C_2_T_x_. Further observation infers that the characteristic peak intensities of CCN at 7.9° and 27.6° in CCN/TCT are slightly lower than that of bare CCN due to the presence of Ti_3_C_2_T_x_. Notably, no apparent diffraction peaks of Ti_3_C_2_T_x_ are observed, which are accredited to the low content and high dispersity of Ti_3_C_2_T_x_,^[^ ^22b^ ^]^ such that the introduction of Ti_3_C_2_T_x_ has a negligible effect on the crystal structure of CCN in the CCN/TCT heterojunction. According to the Scherrer equation, the average crystallite sizes of Ti_3_AlC_2_, Ti_3_C_2_T_x_, g‐C_3_N_4_, CCN, and CCN/TCT are determined to be 39.08, 25.83, 3.74, 10.63, and 11.05 nm, respectively. The larger crystallite sizes observed in CCN and CCN/TCT compared to g‐C_3_N_4_ confirm the enhanced crystallinity resulting from the ionothermal treatment and incorporation of Ti_3_C_2_T_x_, respectively.

FTIR spectra were used to reveal the chemical groups and structures of CCN. As shown in Figure 4f, the FTIR spectra of all the samples are very similar, exemplifying that the g‐C_3_N_4_, CCN, as well as CCN/TCTs share identical chemical groups and structures. The main absorption bands at 3000–3500 cm^−1^ are caused by the ─OH stretching vibration of the surface adsorbed water molecules as well as the vibration of amino groups (─NH_2_ or ─NH) either in terminal or residual parts.^[^ ^36^ ^]^ While the fingerprint signals at 900–1600 cm^−1^ represent the stretching vibration modes of C─N and C═N of the conjugated triazine heterocyclic ring,^[^ ^36^ ^]^ the characteristic band within 700–800 cm^−1^ is assigned to the out‐of‐plane bending vibrations of sp^3^ C─N bonds in heptazine ring.^[^ ^37^ ^]^ Furthermore, the distinctive peaks of CCN and CCN/TCTs at around 2174 cm^−1^ are related to the presence of cyano groups (─C≡N) with high electron‐withdrawing ability, which are originated from the deprotonation of ─C─NH_2_,^[^ ^38^ ^]^ in other words, the partial decomposition of heptazine units during the ionothermal process. This peak is an indication of the formation of a huge amount of surface defects in the CN networks when CCN is prepared via the molten salt method.^[^ ^39^ ^]^ This result is further ascertained by the lowered peak intensities within 3000–3500 cm^−1^ of CCN as compared to g‐C_3_N_4_, revealing that OH^−^ groups released from KOH during the thermal polymerization process will react with ─NH groups to generate cyano groups.^[^ ^40^ ^]^ Besides, the occurrence of a new peak at 990 cm^−1^ is corresponds to the symmetric and asymmetric vibrations of NC_2_ bonds in metal–NC_2_ groups,^[^ ^41^ ^]^ owing to the incorporation of metal ions between the heptazine‐based melon chains.^[^ ^15^, ^41^ ^]^ Moreover, it is noteworthy that there are no distinct peaks in Ti_3_C_2_T_x_ and the frameworks for all the CCN/TCTs are similar to that of CCN, implying that the signals in the hybrids are mainly from CCN. The absence of extra peaks upon incorporating Ti_3_C_2_T_x_ further verifies that the combination of Ti_3_C_2_T_x_ has no effect on the structure of CCN due to the low loading amount of Ti_3_C_2_T_x_. Nonetheless, it is observed that the intensities at 3000–3500 cm^−1^ are lowered after the introduction of Ti_3_C_2_T_x_ into CCN, manifesting that the ─NH_x_ groups on the edges serve as the linkers that form bonds between Ti_3_C_2_T_x_ and CCN.^[^ ^42^ ^]^ Meanwhile, after the hybridization of CCN with Ti_3_C_2_T_x_, a slight shift toward a higher wavenumber is observed in the characteristic peaks of CCN/TCT hybrids (Figure 4 g). This signifies that new chemical bonding is formed between CCN and Ti_3_C_2_T_x_, where the chemical interactions favor the directional electron migration between CCN and Ti_3_C_2_T_x_.^[^ ^10^ ^]^

**Figure 5:**
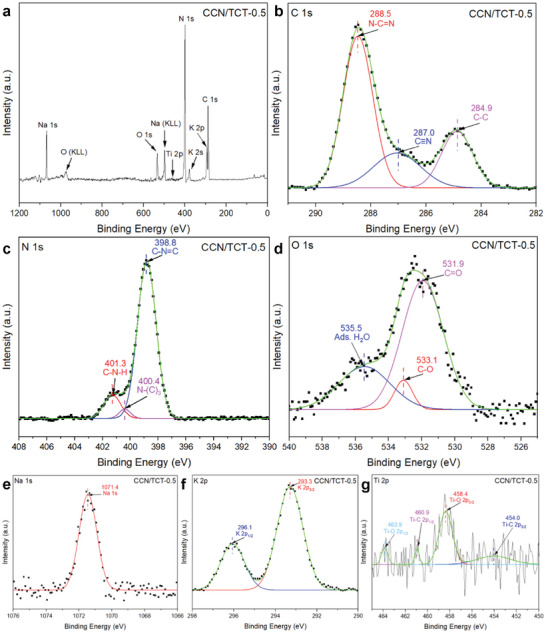
a) XPS survey spectrum and high‐resolution XPS spectra of b) C 1s, c) N 1s, d) O 1s, e) Na 1s, f) K 2p, and g) Ti 2p for CCN/TCT‐0.5 sample.

To further probe the element compositions and chemical states of CCN/TCT, XPS analyses are carried out. As depicted in the XPS survey spectrum (**Figure** **5**a), signals corresponding to the elements C, N, O, Na, K, and Ti are detected, confirming the existence of both CCN and Ti_3_C_2_T_x_ in CCN/TCT hybrids. In the high‐resolution C 1s spectra of CCN/TCT (Figure 5b), three distinct peaks located at 284.9, 287.0, and 288.5 eV are observed, which are assigned to C─C/C═C from carbon contaminants, C≡N/C─NH_x_, and sp^2^‐bonded carbon (N─C═N), respectively.^[^ ^38^ ^]^ In terms of N 1s spectrum (Figure 5c), the signals are deconvoluted into three peaks, in which the peaks at 398.8, 400.4, and 401.3 eV originate from C─N═C, N─(C)_3_, and C─N─H, respectively.^[^ ^30^ ^]^ The results of C 1s and N 1s spectra prove the formation of the heterocyclic structure of g‐C_3_N_4_ heptazine in the CCN/TCT sample. Meanwhile, three oxygen peaks are observed in the O 1s spectrum of CCN/TCT‐0.5 sample (Figure 5d). While the peak located at 531.9 eV is correlated to C═O, the peaks ≈533.1 and 535.5 eV represent the C─O and chemisorbed water molecules on the surface of the sample, respectively.^[^ ^43^ ^]^ Apart from that, XPS investigations verify the presence of Na and K as displayed in Figure 5e,f, respectively. As indicated in Figure 5e, the binding energy of 1071.4 eV is aroused from the Na^+^,^[^ ^33a^ ^]^ revealing the successful incorporation of Na in CCN/TCT hybrids, which corresponds well with the XRD results (Figure 4a). Two peaks are fitted in the K 2p spectrum (Figure 5f) around 293.3 (K 2p_3/2_) and 296.1 eV (K 2p_1/2_) with doublet separation energy of 2.8 eV, demonstrating that K^+^ is introduced into the composite.^[^ ^36^, ^44^ ^]^ Considering the superior conductivity of the metals, the integration of Na and K in the composite is beneficial to the transfer of charge carriers and the enhancement of the photocatalytic activity.^[^ ^36^ ^]^ On the other hand, it is noteworthy that the characteristic peak of TCT is not clearly implied in the XPS survey spectrum (Figure 5a), which is associated with the low loading content of TCT in the hybrid. Nonetheless, the presence of Ti element in CCN/TCT is further verified by the high‐resolution Ti 2p spectrum (Figure 5 g). The Ti 2p spectrum is deconvoluted into four peaks, in which 454.0 and 460.9 eV are related to the Ti 2p_3/2_ and Ti 2p_1/2_ of Ti─C bond, respectively, whereas 458.4 and 463.9 eV belong to Ti 2p_3/2_ and Ti 2p_1/2_ of Ti─O bond, respectively.^[^ ^45^ ^]^

### 3.3 Light Absorption Properties

**Figure 6:**
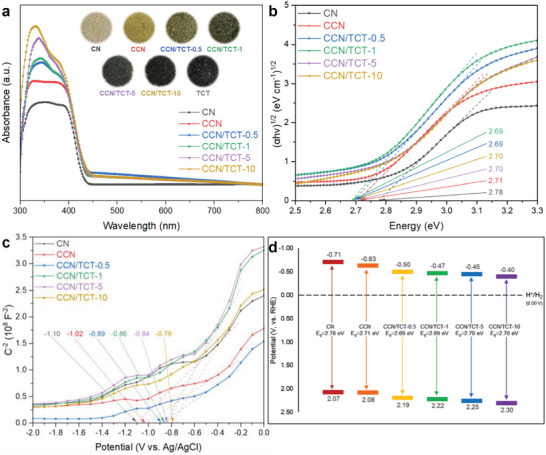
a) UV–vis DRS spectra, b) Tauc plots, c) Mott‐Schottky plots, and d) Schematic band structures of CN, CCN, and a series of CCN/TCT (CCN/TCT‐y, where y is the loading amount of Ti_3_C_2_T_x_) with varying Ti_3_C_2_T_x_ loading amounts. Inset of (a) shows the photographs of the studied catalysts.

The optical properties of the samples were investigated using UV–vis DRS spectra (**Figure** **6**a). Figure 6a indicates the optical absorption of pristine CN, CCN, and a series of CCN/TCTs. Between wavelengths of 300 and 450 nm, both CN and CCN demonstrate high absorption intensities with CCN displaying superior light harvesting capability. It is observed that the absorption edges of CCN experience an obvious red shift in comparison with CN, which is consistent with the slightly darker yellow appearance of the CCN sample. The redshift is attributable to the increased π‐electron delocalization with enhanced structural condensation of CCN.^[^ ^41^ ^]^ At wavelengths beyond 450 nm, both CN and CCN exert lower light absorption ability. Besides the expanded absorption in the visible region, CCN demonstrates elevated light absorption in the UV region, as evidenced by the higher absorption intensities of CCN than that of CN (Figure 6a). As a rule of thumb, the light‐harvesting ability of a conjugated polymer is dependent on its structural rigidity.^[^ ^14^ ^]^ In view of this, the bolstered light absorption of CCN is originated from the increased chain stiffness of the highly crystalline structure of CCN with enhanced interactions between subunits.^[^ ^14^ ^]^

Upon coupling CCN with Ti_3_C_2_T_x_, boosted absorption intensities are observed in the DRS spectra of CCN/TCTs hybrid with respect to those of CN and CCN (Figure 6a). In the wake of increasing Ti_3_C_2_T_x_ blending amount, the color of the hybrid powders gradually darkens and the light‐harvesting ability of the composites becomes stronger,^[^ ^26^ ^]^ as evidenced by the higher absorption peak intensities in Figure 6a. Meanwhile, with the increasing Ti_3_C_2_T_x_ loading, CCN/TCT composites exert a slight red shift as compared to that of CN and CCN, implying greater visible light absorption of the hybrids. This is attributable to the deeper color of the samples, which is beneficial for photocatalytic performance. However, excessive loading of Ti_3_C_2_T_x_ on CCN will block the active sites while exerting a light‐shielding effect, which will adversely impact the photocatalytic performance.^[^ ^10^ ^]^

The band and electronic structures are vital in determining the photocatalytic reaction. As such, the band gap energies (*E*_g_) of CN and CCN are estimated from Tauc plots calculated via the Kubelka–Munk function, where the *E*_g_ values of CN and CCN are determined to be 2.78 and 2.71 eV, respectively (Figure 6b). Overall, CCN exhibits narrower bandgaps in comparison with CN, indicating enhanced light absorption abilities of CCN, which are in line with the observation in Figure 6a. The steeper absorption edges (Figure 6a) and narrower band gaps (Figure 6b) are again stemmed from the enhanced conjugate structure with elevated in‐plane crystallinity.^[^ ^41^ ^]^ On the other hand, the band gap energies are calculated to be 2.69 eV for both CCN/TCT‐0.5 and CCN/TCT‐1; whereas both CCN/TCT‐5 and CCN/TCT‐10 record the same *E*_g_ of 2.70 eV (Figure 6b). The *E*_g_ of all CCN/TCT samples is similar to that of CCN, revealing the fact that there is no substantial effect in altering the band gap energy upon loading Ti_3_C_2_T_x_ onto CCN. Similar results have been demonstrated by Yang's team^[^ ^46^ ^]^ and Tahir's group,^[^ ^47^ ^]^ where the introduction of Ti_3_C_2_T_x_ into g‐C_3_N_4_ did not change the *E*_g_ of g‐C_3_N_4_. Instead, the light absorption ability was enhanced due to the dark color of Ti_3_C_2_T_x_.

To elucidate the energy band structure, Mott‐Schottky analysis was employed to examine the conduction band of the samples. As displayed in Figure 6c, all the CN, CCN, and CCN/TCT samples accord with the typical characteristics of *n*‐type semiconductors, as evidenced by their positive slopes, where the flat band potentials are acquired and converted into their corresponding conduction band potential (*E*_CB_).^[^ ^22^, ^46^ ^]^ From Figure 6c, the flat band potential of CN and CCN are measured to be −1.10 and −1.02 eV (versus Ag/AgCl, pH = 6.7), respectively, which are equivalent to −0.51 and −0.43 eV (vs RHE), according to the conversion formula of Ag/AgCl electrode potential to reversible hydrogen electrode potential,

(2) $$E_{RHE}=E_{Ag/AgCl}+0.059\times pH+E_{Ag/AgCl}^{\theta }$$

Where pH = 6.7 and $E_{Ag/AgCl}^{\theta }=0.197\;V$ at 25 °C.^[^ ^48^ ^]^

Generally, the conduction band potential of *n*‐type semiconductors is 0.1 to 0.3 V more negative than the corresponding flat band potential (*E*_fb_) due to the absence of exact doping concentration.^[^ ^49^ ^]^ Therefore, the *E*_CB_ of CN and CCN are calculated to be −0.71 and −0.63 V (versus RHE), respectively. The conduction band potentials are similar to that found in literature, where the E_CB_ of g‐C_3_N_4_ has been reported to be ranging from −0.56 to −1.29 V (versus RHE).^[^ ^26^, ^46^, ^49^, ^50^ ^]^ The formula *E_VB_* = *E_CB_*  + *E_g_* is used to compute the valence band potentials (*E*_VB_) of the photocatalysts. Figure 6d depicts the schematic band structures of CN, CCN, and all the CCN/TCT samples. As observed, the CCN prepared in the presence of molten salts present a positive shift in the E_CB_ as compared to pristine CN, which is consistent with the results reported by Zhang's group,^[^ ^51^ ^]^ manifesting the higher reductive ability of photoexcited electrons of CCN. Meanwhile, the *E*_VB_ of CCNs shifts to more positive values as compared to CN, signifying the elevated oxidative ability of the photogenerated holes in driving oxidation reactions.

The effect of Ti_3_C_2_T_x_ loading on the band structures of CCN was investigated as well. Based on the *E*_g_ and *E*_CB_ obtained from Figure 6b,c, respectively, the energy band positions of CCN/TCT composites are illustrated in Figure 6d. Notably, the *E*_CB_ of CCN/TCT hybrids displays a positive shift compared to pristine CCN (Figure 6c), with increasing Ti_3_C_2_T_x_ loading. This shift implies a reduction in the energy barrier for hydrogen reduction, facilitating electron transfer and augmenting the hydrogen evolution reaction. Concurrently, the *E*_VB_ of CCN/TCTs also becomes more positive with the addition of Ti_3_C_2_T_x_, suggesting enhanced suitability for driving oxidative reactions. Overall, these findings suggest a synergistic effect between CCN and Ti_3_C_2_T_x_, with Ti_3_C_2_T_x_ serving as an electron reservoir and boosting efficient charge transfer and separation. This synergistic effect is further supported by the discussion in Section 3.1. (Figure 3h), where the formation of a commendable interface between CCN and Ti_3_C_2_T_x_ is observed. Specifically, when CCN and Ti_3_C_2_T_x_ come into intimate contact, the photogenerated electrons transfer from CCN to Ti_3_C_2_T_x_, establishing a favorable interface at the heterojunction. This interface enhances charge carrier transportation and separation, ultimately contributing to improved photocatalytic activity.^[^ ^22b^ ^]^

### 3.4 Photoelectrochemical Properties

**Figure 7:**
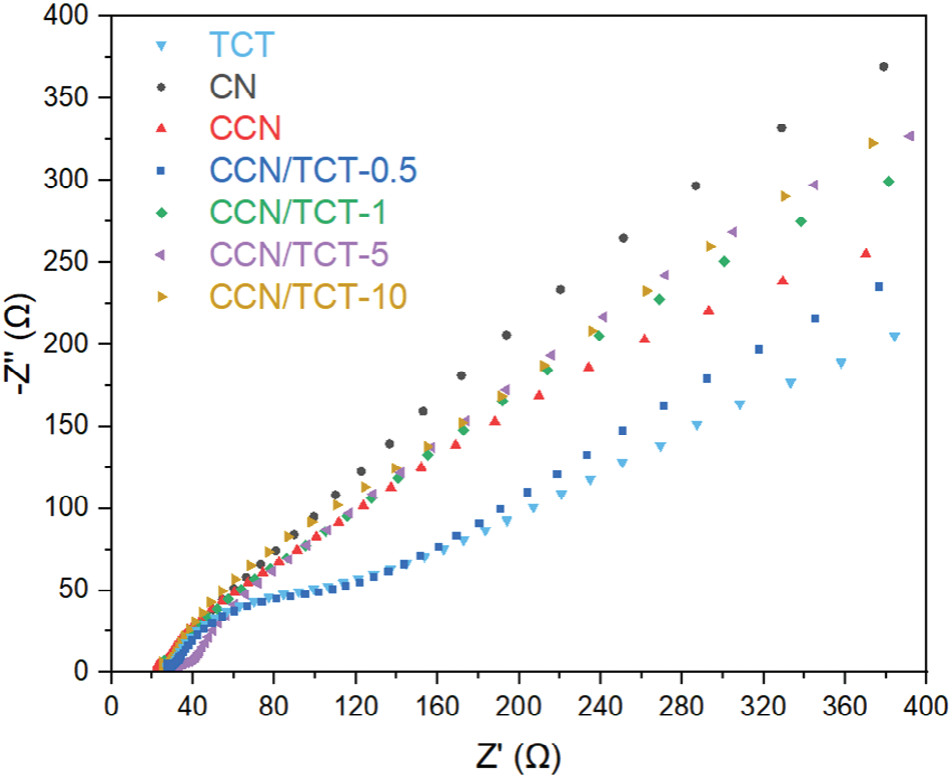
EIS Nyquist plots of the as‐prepared samples of TCT, CN, CCN, and CCN coupled with varying Ti_3_C_2_T_x_ amounts.

To gain insights into the effect of crystallinity in enhancing the charge separation performance of CN, electrochemical tests were conducted. As shown in **Figure** **7**, CCN presents a smaller arc radius than that of CN, signifying that the in‐plane crystallinity of CCN, as verified in the XRD (Figure 4a) and FTIR plots (Figure 4f), is propitious to lowering the charge transfer resistance and bolstering charge mobility within the photocatalysts. Besides, the K^+^ intercalated between the melon chains in CCN functions as an electron bridge that facilitates the carrier transfer between adjacent molecular frameworks, such that the charge transfer efficiency in CCN is superior to that of CN.^[^ ^14^ ^]^ As such, the smaller arc radius of CCN (Figure 7) suggests its potential for enhanced photocatalytic performance, which will be further affirmed in Section 3.5.

On the other hand, Ti_3_C_2_T_x_ exerts the smallest radius (Figure 7) owing to its remarkable electronic conductivity.^[^ ^18^ ^]^ Upon coupling with Ti_3_C_2_T_x_, CCN/TCT‐0.5 displays a smaller EIS radius compared to pristine CCN, demonstrating that an appropriate loading of Ti_3_C_2_T_x_ contributes to reduced charge transfer resistance and heightened interfacial charge transport ability, favoring efficient separation of photomotivated charge carriers. These results reveal that the synergistic interaction of Ti_3_C_2_T_x_, acting as the electron sink, and CCN, serving as the semiconductor photocatalysts, in the heterostructure enables rapid interfacial carrier migration and realizes excellent spatial segregation efficiency of charges, thereby facilitating the photocatalytic reaction.^[^ ^22a^ ^]^ Although metallic Ti_3_C_2_T_x_ brings about vast electron mobility and magnificent conductivity of CCN/TCT, it should be noted that excessive loading of Ti_3_C_2_T_x_ cocatalyst creates recombination centers and induces light shielding effect, which hampers the charge separation and photocatalytic activity.^[^ ^26^ ^]^ From Figure 7, it is observed that the interfacial charge transfer efficiency of the composites decreases when the loading amount of Ti_3_C_2_T_x_ exceeds 0.5 wt.%. In particular, CCN/TCT with 0.5 wt.% Ti_3_C_2_T_x_ exhibits the smallest impedance radius, indicating that this loading amount is optimal for promoting charge separation and migration in the hybrid system. Beyond this value, a further increase in the cocatalyst content in the composite leads to weaker charge segregation performance, as evidenced by the expanding radius of other CCN/TCTs with increasing Ti_3_C_2_T_x_ amount in Figure 7. Overloading semiconductor photocatalysts with cocatalysts leads to the generation of recombination centers and brings about light‐screening effect, ultimately harming the photocatalytic capacity.^[^ ^10^ ^]^

### 3.5 Photocatalytic Activity

**Figure 8:**
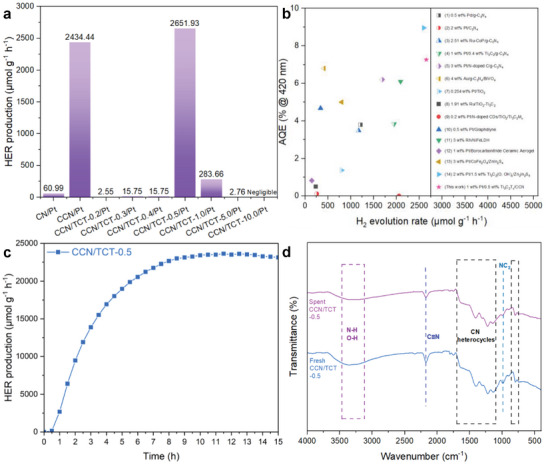
a) Hydrogen evolution rate of CN, CCN, and CCN/TCT‐y (where y is the loading amount of Ti_3_C_2_T_x_) in a single H_2_ reaction over 4 h reaction time. b) Comparison between different photocatalysts and CCN/TCT‐0.5/Pt in terms of H_2_ evolution rate and AQE. (Ref.: 1)^[^ ^54^ ^]^, 2)^[^ ^55^ ^]^, 3)^[^ ^56^ ^]^, 4)^[^ ^23^ ^]^, 5)^[^ ^57^ ^]^, 6)^[^ ^58^ ^]^, 7)^[^ ^59^ ^]^, 8)^[^ ^60^ ^]^, 9)^[^ ^61^ ^]^, 10)^[^ ^62^ ^]^, 11)^[^ ^63^ ^]^, 12)^[^ ^64^ ^]^, 13)^[^ ^65^ ^]^, 14)^[^ ^66^ ^]^) c) Photocatalytic hydrogen production rate as a function of illumination time over CCN/TCT‐0.5. d) FTIR spectra of CCN/TCT‐0.5 before and after hydrogen production stability test.

The photocatalytic H_2_ evolution reaction was performed in a TEOA aqueous solution composed of 6 mL TEOA, 54 mL DI water, 0.534 mL H_2_PtCl_6_•6H_2_O, and 20 mg photocatalysts, using a Xe‐lamp as the light source (*λ* > 420 nm). **Figure** **8**a compares the photocatalytic H_2_ formation of CN, CCN, and a series of CCN/TCTs over the course of 4 h of reaction time. Inspiringly, CCN displays impressive activity toward hydrogen production with a decent amount of 2434.44 µmol g^−1^ h^−1^, which is around 39.92 times higher than that of pristine CN. The improvement of photocatalytic performance of CCN as compared to CN is assigned to its sufficient condensation of the conjugated framework, as the unreacted amino groups that act as recombination sites are significantly reduced in the CN framework.^[^ ^43^ ^]^ This accords with the narrower peaks of (100) and (002) planes in the XRD curves (Figure 4a) and the lowered peak intensities of ─NH groups in FTIR plots of CN and CCN (Figure 4f). The ameliorated crystallinity of CCN facilitates charge separation and transfer,^[^ ^53^ ^]^ such that more highly reactive electron‐hole pairs are able to partake in the surface reactions. Meanwhile, the suitable band position of CCN (Figure 6d) enables it to harvest a wide range of visible light to motivate more electrons and accelerate charge segregation to drive the photocatalytic reductive reaction. On the other hand, the broadband energy (Figure 6d) and low crystallinity of CN restrict the efficient formation and segregation of photoexcited charge,^[^ ^53b^ ^]^ which leads to low hydrogen production. As a result, CCN, which exerts excellent hydrogen formation efficiency (Figure 8a), presents a better performance in the H_2_ evolution reaction as compared to that of CN. This result corresponds well with the smaller arc radius of CCN as compared to that of CN in the EIS plots (Figure 7) as discussed in Section 3.4.

To further enhance the performance of CCN in light‐driven hydrogen generation, Ti_3_C_2_T_x_ is integrated into CCN to form CCN/TCT hybrids. The photocatalytic hydrogen evolution performance of the as‐prepared CCN/TCTs has been assessed with different loading amounts of Ti_3_C_2_T_x_ to identify the optimal loading contents under visible light illumination with TEOA as hole scavenger and Pt as the cocatalysts (Figure 8a). Compared to CCN/Pt, CCN/TCT‐0.5/Pt demonstrates ameliorated H_2_ generation rates. When pure CCN is used as photocatalysts, a lower H_2_ yield of 2434.44 µmol g^−1^ h^−1^ is attained due to the low separation efficiency of photoinduced charge carriers.^[^ ^67^ ^]^ This limited separation results in only a fraction of effective electrons being capable of activating the conversion of protons into H_2_. When 0.5 wt.% Ti_3_C_2_T_x_ is anchored with CCN/Pt, a magnificent H_2_ production rate of 2651.93 µmol g^−1^ h^−1^ is achieved (Figure 8a), where the augmented performance arises from the introduction of Ti_3_C_2_T_x_ that aids in charge carrier segregation and serves as reactive sites.^[^ ^68^ ^]^ Meanwhile, a high AQE of 7.26% (420 nm) is achieved by CCN/TCT‐0.5/Pt, manifesting the impressive photoactivity of CCN/TCT‐0.5/Pt. The synergistic effect of the superior conductivity of Ti_3_C_2_T_x_, abundant active sites offered by Ti_3_C_2_T_x_ and Pt, and the close interfacial contact developed between CCN and Ti_3_C_2_T_x_, as evidenced by the tight interface in the TEM images (Figure 3g,h), is conducive to facilitating the charge carrier separation and migration within the composite.^[^ ^69^ ^]^ Due to the high conductivity of Ti_3_C_2_T_x_, the photogenerated electrons from the CCN tend to flow to Ti_3_C_2_T_x_. On the other hand, due to the difference in Fermi levels between Ti_3_C_2_T_x_ and Pt, the electrons held by Ti_3_C_2_T_x_ are prone to transferring to Pt, achieving a lower energy state. It has been reported that the presence of Pt would facilitate the transfer of electrons accumulated in Ti_3_C_2_T_x_ to Pt, where this additional electron transfer pathway further intensifies the charge separation ability of the composite.^[^ ^70^ ^]^ Once the electrons reach Pt, they partake in a hydrogen evolution reaction; whereas Ti_3_C_2_T_x_ continues to capture and transfer electrons, ensuring a continuous supply of electrons for photocatalytic reactions occurring at the Pt sites. Aside from the above‐mentioned orientation, Pt and Ti_3_C_2_T_x_ may distribute separately, where the photomotivated electrons directly transfer into the cocatalysts and store inside them, giving rise to the enhancement in charge segregation. Thus, the presence of Pt and Ti_3_C_2_T_x_ in the composites enables the spatial isolation of photogenerated electron‐hole pairs, preventing the undesired charge recombination in the photocatalysts, thereby allowing efficient charge separation. In the context of hydrogen production, both Ti_3_C_2_T_x_ and Pt provide adsorption and active sites for hydrogen reduction reactions due to their affinity toward protons. The captured electrons in both cocatalysts reduce protons to hydrogen gas at the active sites. Therefore, while the incorporation of Ti_3_C_2_T_x_ as an electron sink fosters the charge segregation, the collaborative synergy between Ti_3_C_2_T_x_ and Pt dual cocatalysts that provides increased surface reactive sites further endows CCN/TCT‐0.5/Pt with superior photoactivity.

However, increasing the loading amount of Ti_3_C_2_T_x_ beyond 0.5 wt.% brings adverse impacts on the hydrogen generation activity (Figure 8a), where the production of H_2_ reduces with increasing Ti_3_C_2_T_x_ amount. This result aligns with the reported charge separation performance of the composites shown in Figure 7, where CCN/TCT‐0.5 exerts the second smallest arc radius, signifying superior charge segregation efficiency; whilst other CCN/TCTs (CCN/TCT‐y, *y* = 1, 5, 10) show an expanding arc radius with increasing Ti_3_C_2_T_x_ loading, which brings about reduced charge separation ability, and hence lower H_2_ yield. This phenomenon is associated with the excessive loading of cocatalysts, leading to the creation of recombination centers and light shielding effect.^[^ ^10^ ^]^ Among all the CCN/TCT/Pt composites, the hybrid integrated with 0.5 wt.% Ti_3_C_2_T_x_ demonstrates the highest H_2_ production rate, signifying that 0.5 wt.% is the optimal loading amount of Ti_3_C_2_T_x_ cocatalysts on CCN in photocatalytic HER. Meanwhile, the decline in efficiency of CCN/TCT/Pt composites with loadings ranging from 0.2 to 0.4 wt.% can be attributed to several factors, such as possible aggregation of Ti_3_C_2_T_x_ particles within the CCN matrix and possible inhibition of reaction by Ti_3_C_2_T_x_ at lower loadings. These limitations hinder the catalytic activity of CCN/Pt composites loaded with 0.2 to 0.4 wt.% Ti_3_C_2_T_x_, leading to diminished production rates compared to pure CCN/Pt and CCN/TCT‐0.5/Pt. As a whole, the superior performance of CCN/TCT‐0.5/Pt underscores the importance of optimizing Ti_3_C_2_T_x_ loading amounts for maximizing the catalytic performance of CCN composites. Further research is essential to elucidate the underlying mechanisms governing the interactions between CCN and Ti_3_C_2_T_x_ to unlock the full potential of these composites in various applications.

In Figure 8b, a comparison of various catalysts for hydrogen evolution is presented. Generally, precious metals including platinum, ruthenium (Ru), rhodium (Rh), palladium (Pd), and gold (Au) are used as cocatalysts on semiconductor photocatalysts to address the limitations of photocatalysts. It is important to note that Ru, Rh, Pd, and Au often come with higher price tags than Pt due to limited availability and higher production costs. As illustrated in Figure 8b, catalysts like Pd/g‐C_3_N_4_,^[^ ^54^ ^]^ Au/g‐C_3_N_4_/BiVO_4_,^[^ ^58^ ^]^ Ru‐CoP/g‐C_3_N_4_,^[^ ^56^ ^]^ and Rh/NiFeLDH,^[^ ^63^ ^]^ which comprise of these more expensive noble metals, are less economically favorable. Aside from this, the loading amount of Pt varies among different photocatalytic systems. Some systems with low Pt loading, such as 0.254 wt.% Pt/TiO_2_^[^ ^59^ ^]^ and 0.5 wt.% Pt/Graphdiyne,^[^ ^62^ ^]^ demonstrated lower performance levels in terms of H_2_ generation and AQE as compared to the optimal sample in this work (1 wt.% Pt/0.5 wt.% Ti_3_C_2_T_x_/CCN). Conversely, systems like N‐doped VC/C_3_N_4_ coupled with 2 wt.% Pt displayed a hydrogen production rate of 766 µmol g^−1^ h^−1^ and an AQE of 0.2% (420 nm),^[^ ^55^ ^]^ while 3 wt.% Pt/CoFe_2_O_4_/ZnIn_2_S_4_ exerted remarkable HER performance with a hydrogen evolution efficiency and an AQE of 800 µmol g^−1^ h^−1^ and 5% (420 nm),^[^ ^65^ ^]^ respectively. Despite ameliorating the photocatalytic activity, these high Pt loading systems (with loading amounts > 1 wt.%) incur increased costs, limiting their viability for large‐scale applications. It is noteworthy that Pt/Ti_3_C_2_(O, OH)_x_/Zn_2_In_2_S_5_^[^ ^66^ ^]^ showed a comparable HER efficiency and AQE (2596.8 µmol g^−1^ h^−1^, 8.96%@420 nm) to the optimal catalyst in this work, but the higher loading amount of Pt and MXene required in the Pt/Ti_3_C_2_(O,OH)_x_/Zn_2_In_2_S_5_ hybrids renders the system less economically feasible. Therefore, considering both cost‐effectiveness and performance, the catalyst developed in this work, CCN loaded with 1 wt.% Pt and 0.5 wt.% Ti_3_C_2_T_x_ emerges as a superior or comparable option to the catalysts shown in Figure 8b. It delivers an impressive hydrogen production rate of 2651.93 µmol g^−1^ h^−1^ and a commendable AQE of 7.26% (420 nm). Overall, the combination of its exceptional photocatalytic performance and cost‐effectiveness positions it as a promising choice for photocatalytic hydrogen evolution applications.

The durability of a photocatalyst is a crucial parameter in the potential application of photocatalytic hydrogen production reaction. To evaluate the long‐term stability in a continuous reaction system, it is beneficial to conduct long‐run activity tests rather than cyclic tests. A stable photocatalyst should maintain a constant rate of hydrogen evolution, while any significant deviation in hydrogen generation upon reaching a steady state indicates a decline in photocatalyst activity. In light of this, the long‐term stability test result of the representative CCN/TCT‐0.5 is presented in Figure 8c. Distinctly, the CCN/TCT‐0.5 photocatalyst reaches a steady state of photocatalytic H_2_ generation (≈23 000 µmol g^−1^ h^−1^) after 8 h of reactions. Upon that, the reaction rates remain steady without apparent change in hydrogen evolution at extended illumination times. This result signifies that the CCN/TCT‐0.5 photocatalyst possesses marvelous stable performance even under long‐run working conditions, highlighting its potential application in continuous processes suitable for commercialization. Additionally, FTIR measurement was utilized to characterize the spent CCN/TCT‐0.5 after photocatalytic HER (Figure 8d). It is found that the overall FTIR spectra of both fresh and spent CCN/TCT‐0.5 are similar, with little to negligible change between them, thus further manifesting its stable structure after photocatalytic reaction.

To further demonstrate the potential of CCN/TCT‐0.5/Pt, the photocatalytic performance of carbon nitride and its hybrids toward the H_2_ evolution reaction is being compared with that of the optimal catalysts in this work (**Table** **1**). As observed, generally, g‐C_3_N_4_/Ti_3_C_2_T_x_ hybrids without Pt as cocatalysts exerted subpar photocatalytic performance to that of carbon nitride‐MXene system with incorporated Pt. For instance, p‐g‐C_3_N_4_/Ti_3_C_2_T_x_ prepared by Xu et al.^[^ ^22c^ ^]^ achieved a H_2_ evolution rate of 727 µmol g^−1^ h^−1^ whereas the p‐g‐C_3_N_4_/Ti_3_C_2_T_x_ hybrid reported by Kang and coworkers^[^ ^22b^ ^]^ attained a high H_2_ production rate of 982.2 µmol g^−1^ h^−1^. Similar results were observed for g‐C_3_N_4_/Ti_3_C_2_ synthesized by Dong's team^[^ ^22a^ ^]^ and Li's group.^[^ ^22^, ^50^ ^]^ It should be noted that the g‐C_3_N_4_/Ti_3_C_2_/Pt prepared by Liu and coworkers^[^ ^23^ ^]^ exhibited a relatively high photocatalytic activity, recording a H_2_ production performance of 1948 µmol g^−1^ h^−1^ and an AQE of 3.83% (420 nm). The 3D interconnected structure as well as the presence of Ti_3_C_2_ and Pt as cocatalysts contributed to the ameliorated photoactivity of g‐C_3_N_4_/Ti_3_C_2_/Pt. The composite with a 3D interconnected structure is expected to have elevated surface area as compared to that of the CCN in the current work, which is usually beneficial for the ameliorated photoactivity as the reactants have a higher possibility to contact with the catalysts. Despite the fact that the CCN generated in this study may be inferior to that of the 3D g‐C_3_N_4_ reported by Liu's team, CCN/TCT‐0.5/Pt in this work still outperforms g‐C_3_N_4_/Ti_3_C_2_/Pt in terms of both H_2_ evolution rate (2651.93 µmol g^−1^ h^−1^) and AQE (7.26%, 420 nm), which is ascribed to the synergistic effect of CCN with high crystallinity and Ti_3_C_2_/Pt dual cocatalysts that offers ample active sites and promotes charge separation. It is worth mentioning that each g‐C_3_N_4_/Ti_3_C_2_ hybrid possessed excellent stability (>15 h), indicating the stability of carbon nitride/MXene composites in general. However, it should be noted that the stability of other g‐C_3_N_4_/Ti_3_C_2_ composites, aside from the catalyst from this study, was evaluated based on cyclic tests, which focus on the reusability of catalysts, rather than long‐term stability tests. This limitation hinders the direct comparison of the catalysts’ stability in long‐run activity. Overall, the performance of CCN/TCT‐0.5/Pt is superior to that of other similar hybrids, showcasing the vast potential of CCN/TCT to serve as an efficient system in light‐driven catalysis processes.

**Table 1:** Comparison of photocatalytic performance of g‐C_3_N_4_/Ti_3_C_2_T_x_ in H_2_ evolution reaction.

Photocatalyst	Sacrificial Agent	Light Source (Wavelength)	H_2_ Evolution Rate (µmol g^−1^ h^−1^)	AQE (Wavelength)	Stability (h)	Reference
p‐g‐C_3_N_4_/Ti_3_C_2_T_x_	10 vol% Triethanolamine	300 W xenon lamp (λ > 420 nm)	727	8.6% (420 nm)	>15	Xu et al.^[^ ^22c^ ^]^
p‐g‐C_3_N_4_/Ti_3_C_2_T_x_	10 vol% Triethanolamine	300 W xenon lamp (NA)	982.2	NA	>15	Kang et al.^[^ ^22b^ ^]^
g‐C_3_N_4_/Ti_3_C_2_T_x_	20 vol% Triethanolamine	300 W xenon lamp (λ > 420 nm)	534	1.61% (420 nm)	>60	Dong et al.^[^ ^22a^ ^]^
2D/3D g‐C_3_N_4_/Ti_3_C_2_	10 vol% Triethanolamine	300 W xenon lamp (λ > 420 nm)	727	NA	>15	Li et al.^[^ ^50a^ ^]^
3D/2D g‐C_3_N_4_/Ti_3_C_2_/Pt	10 vol% Triethanolamine	300 W xenon lamp (λ > 420 nm)	1948	3.83% (420 nm)	>15	Liu et al.^[^ ^23^ ^]^
Crystalline carbon nitride/Ti_3_C_2_/Pt	10 vol% Triethanolamine	300 W xenon lamp (λ > 420 nm)	2651.93	7.26% (420 nm)	>15	This work

### 3.6 Possible Charge Transport Mechanism

**Figure 9:**
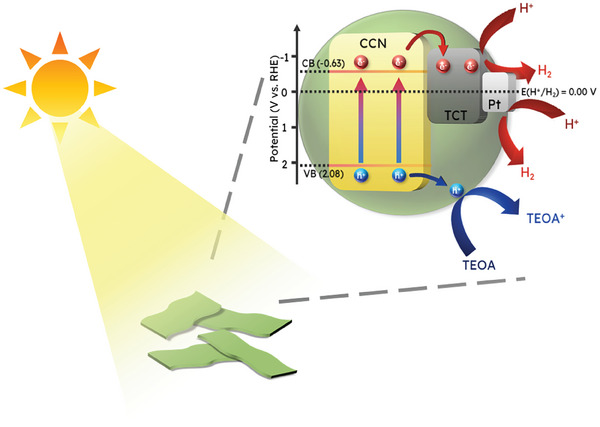
Postulated charge transport mechanism over CCN/TCT/Pt upon light illumination.

In light of the aforementioned discussion, the overall mechanism proposed for the photocatalytic H_2_ evolution over CCN/TCT/Pt heterostructure is illustrated in **Figure** **9**. Benefitting from the extraordinary conductivity as well as the more negative E_F_ of Ti_3_C_2_T_x_, when CCN and Ti_3_C_2_T_x_ come into contact, the electrons in CCN transfer to Ti_3_C_2_T_x_ for equilibrating the Fermi level between the two materials.^[^ ^70^ ^]^ The same goes for Pt. Under visible light illumination, the electrons in CCN are excited and jump from the VB to the CB of CCN, leaving an equivalent number of holes in the VB. The photogenerated electrons fleetly migrate across the intimate heterointerface to Ti_3_C_2_T_x_ and Pt, which serve as the electron reservoirs. While the crystalline structure of CCN hinders the recombination of isolated photomotivated charges during the transportation process,^[^ ^48^ ^]^ it should be noted that the CCN with high crystallinity still has unavoidable defects on it, where during the charge transport and migration process, some electrons and holes will recombine in the defects, resulting in limited charges partaking in the photocatalytic reactions. Meanwhile, the presence of electron reservoirs greatly restrains the photoactivated electrons migrated to the cocatalysts from back‐flowing, hence enabling an elevated amount of strong reductive electrons to participate in the photocatalytic HER.^[^ ^42^ ^]^ Simultaneously, the photoexcited holes with strong oxidative capability that are accumulated in the VB of CCN partake in the oxidation of the TEOA sacrificial agent. As a result, the CCN/TCT‐0.5/Pt exhibits a bolstered performance for light‐driven H_2_ evolution.

## 4 Conclusion

In summary, the CCN/TCT hybrids with ameliorated crystallinity and charge transport capacity have been successfully prepared using a one‐step calcination process assisted by alkaline salts, followed by a freeze–drying method. The effect of crystallinity in charge transfer and separation efficiency as well as the photocatalytic performance in the H_2_ evolution reaction are evaluated, in which the crystallinity of CCN is ascertained by XRD, FTIR, and XPS. Overall, CCN exerted superior photocatalytic activities than CN, as the more ordered arrangement of the heptazine‐based melon chains has lesser reintegration centers, thereby enabling fleet electron‐hole pair separation and transfer, which is conducive to bolstering the surface redox reactions. On the basis of CCN, a series of CCN/TCT has been successfully fabricated by employing the freeze–drying method, where the interaction between CCN and Ti_3_C_2_T_x_ was governed by the van der Waals force. CCN/TCT‐0.5/Pt achieved an elevated H_2_ evolution rate of 2651.93 µmol g^−1^ h^−1^, which was ≈43.5 and ≈1.1 times higher than that of CN/Pt and CCN/Pt, respectively. Simultaneously, it attained an appreciable AQE of 7.26% (420 nm) and it was steady under long‐run working conditions (>15 h). The distinctly boosted photoactivity originated from the synergistic effects of the highly crystalline structure of CCN, distinguished conductivity of Ti_3_C_2_T_x_, abundant active sites offered by Ti_3_C_2_T_x_ and Pt, as well as the intimate heterojunction that was constructed at the interface between CCN and Ti_3_C_2_T_x_. This work sheds light on the improvement of photocatalytic performance via cocatalyst loading and provides new insights into the design of highly efficient photocatalysts using crystalline carbon nitride and MXene.

## Conflict of Interest

The authors declare no conflict of interest.

## Data Availability

The data that support the findings of this study are available from the corresponding author upon reasonable request.
